# ^225^Ac/^89^Zr-Labeled N4MU01 Radioimmunoconjugates as Theranostics Against Nectin-4–Positive Triple-Negative Breast Cancer

**DOI:** 10.2967/jnumed.124.268387

**Published:** 2025-04

**Authors:** Hanan Babeker, Fabrice Ngoh Njotu, Jessica Pougoue Ketchemen, Alissar Monzer, Anjong Florence Tikum, Alireza Doroudi, Emmanuel Nwangele, Maruti Uppalapati, Humphrey Fonge

**Affiliations:** 1 Department of Medical Imaging, College of Medicine, University of Saskatchewan, Saskatoon, Saskatchewan, Canada;; 2 Department of Pathology and Laboratory Medicine, College of Medicine, University of Saskatchewan, Saskatoon, Saskatchewan, Canada;; 3 Axe Oncologie, Centre de Recherche du CHU de Québec-Université Laval, Québec, Quebec, Canada; and; 4 Faculté de Pharmacie, Université Laval, Ferdinand Andry Pavillon, Québec City, Quebec, Canada

**Keywords:** nectin-4, triple-negative breast cancer, human anti–nectin-4 antibody, radioimmunoconjugates, PET/CT, targeted α-particle therapy

## Abstract

Nectin-4 is an overexpressed biomarker in 60%–70% of triple-negative breast cancer (TNBC) cases and an ideal target for radiotherapy and PET imaging. In this study, theranostic radioimmunoconjugates were developed using a fully human anti–nectin-4 antibody (N4MU01). The imaging properties and therapeutic effectiveness of the radioimmunoconjugates were evaluated using TNBC models. **Methods:** N4MU01 was radiolabeled with ^89^Zr and ^225^Ac for imaging and radiotherapy, respectively, using TNBC xenograft and syngeneic models. Biodistribution and PET imaging of the [^89^Zr]Zr-deferoxamine (DFO)-N4MU01 radioimmunoconjugate was studied in mice bearing nectin-4–positive xenografts. Dosimetry and toxicity of [^225^Ac]Ac-Macropa-N4MU01 were studied in naïve BALB/c mice, and the therapeutic efficacy was evaluated with two doses of 13 or two doses of 18.6 kBq, administered 10 d apart in athymic BALB/c nude mice bearing either a human TNBC MDA-MB-468 xenograft or a human nectin-4–transfected 4T1 (4T1._nectin-4_) syngeneic allograft. **Results:** The pharmacokinetic profile of the [^89^Zr]Zr-DFO-N4MU01 radioimmunoconjugate showed biphasic distribution with a moderate elimination half-life of 63 h. PET imaging and biodistribution of [^89^Zr]Zr-DFO-N4MU01 in mice bearing the MDA-MB-468 xenograft showed high tumor uptake of 13.2 ± 1.12 percent injected activity per gram at 120 h. [^225^Ac]Ac-Macropa-N4MU01 was effectively internalized in MDA-MB-468 and was cytotoxic to the cells with a 50% inhibition concentration of 1.2 kBq/mL. Toxicity studies revealed that 15 kBq of [^225^Ac]Ac-Macropa-N4MU01 was generally well tolerated, as indicated by hematologic, blood chemistry, and histopathologic analysis. Mice bearing MDA-MB-468 and 4T1._nectin-4_ xenografts treated with 13 kBq of [^225^Ac]Ac-Macropa-N4MU01 had 100% (6/6) and 83.3% (5/6) complete tumor remissions, respectively. **Conclusion:** The specific tumor uptake and remarkable effectiveness against aggressive TNBC tumors are very promising and warrant the clinical development of N4MU01 radioimmunoconjugates.

## Visual Abstract

**Figure:**
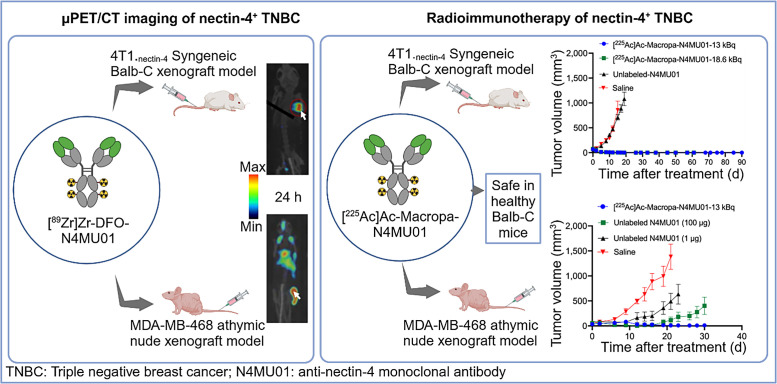

Breast cancer is the most common cancer in women, with 1 in 8 women likely to be diagnosed with the disease in their lifetime. Triple-negative breast cancer (TNBC) represents 10%–20% of invasive breast cancer and has been associated with race, deprivation status, younger age at diagnosis, more advanced disease stage at diagnosis, higher grade, high mitotic indices, family history of breast cancer, and BRCA1 mutations and is aggressive with a poor prognosis (1). Taxanes and anthracyclines, alone or in combination, constitute the first line of treatment for metastatic breast cancer with response rates of 38% and 46% for single-agent and combination-based therapies, respectively (2). However, de novo resistance to taxanes is common in over 50% of patients, and most patients acquire resistance (2). Anti-TROP2 antibody–drug conjugate (ADC) sacituzumab govitecan is the only antibody-based therapeutic recently approved for the treatment of TNBC (3). Despite the promising outcome using sacituzumab govitecan, novel approaches are required.

Poliovirus receptor–related protein 4/nectin-4 is uniquely overexpressed in most cancers of epithelial origin (including gastric, breast, lung, pancreatic, ovarian, bladder, and esophageal) but not in nonmalignant or healthy tissues (4,5). Large clinical studies have consistently confirmed that 60%–70% of all TNBC overexpress nectin-4 (5–7). Immunohistochemical analysis of a panel of 294 healthy tissue specimens from 36 healthy human organs showed weak homogeneous staining mainly in human skin keratinocytes, skin appendages (sweat glands and hair follicles), transitional epithelium of the bladder, salivary gland (ducts), esophagus, breast, and stomach, with no staining from other tissues (7). Therefore, nectin-4 is an excellent biomarker for targeted radioimmunotherapy of TNBC.

Enfortumab vedotin is a fully human anti–nectin-4 ADC that is approved for the treatment of nectin-4–positive advanced urothelial cancer (8). In preclinical studies, enfortumab vedotin was effective in mice bearing nectin-4–positive TNBC tumors and is currently in phase II trials in patients with advanced metastatic solid tumors including TNBC (EV-202 trial) (6). In mice bearing nectin-4–positive TNBC xenografts, complete remission was seen in most tumors, but unfortunately, all regrew after periods of no treatment. Although these recurrent tumors were sensitive to the drug, the same manner of tumor regrowth was observed after subsequent secession of treatment (6). Cabaud et al. (9) showed preclinically that the mechanism of resistance to the anti–nectin-4 ADC was due to the expression of the ABCB1 gene, encoding the multi-drug-resistant protein/P-glycoprotein, associated with focal gene amplification and high protein expression. This is a common mechanism of resistance of most ADCs (9–11).

Unlike ADCs, resistance to α-particle therapeutics has never been observed (12,13). ^225^Ac is an ideal α-particle emitter because of its decay characteristics. We have previously developed a fully human anti–nectin-4 antibody (N4MU01) using phage display (parallel manuscript). In this work, a N4MU01 theranostic (therapeutic and diagnostic) was developed. [^89^Zr]Zr-deferoxamine (DFO)-N4MU01 and [^225^Ac]Ac-Macropa-N4MU01 were developed as PET imaging and radioimmunotherapeutic agents, respectively, against nectin-4–positive TNBC.

## MATERIALS AND METHODS

### Cell Lines and Xenografts

Human breast cancer cell lines MDA-MB-468 and MCF-7, which express nectin-4, and MDA-MB-231, known for nectin-4–negative expression, were purchased from American Type Culture Collection. For a syngeneic mouse model, the mouse breast cancer cell line 4T1 was purchased from American Type Culture Collection and transfected with the full-length nectin-4 protein (4T1._nectin-4_). Description of cell lines and xenografts is available in the supplemental materials at http://jnm.snmjournals.org (14).

### In Vitro Characterization of N4MU01

The binding affinity and specificity of N4MU01 were evaluated by flow cytometry using nectin-4–expressing breast cancer cells MDA-MB-468, MCF-7, 4T1._nectin-4_, and nectin-4–negative MDA-MB-231 as described previously (15). The internalization of N4MU01 was studied for 48 h using the Incucyte live-cell imaging system (Sartorius) as described previously (15) (details of in vitro characterization are in the supplemental materials).

### Conjugation with Bifunctional Chelators and Radiolabeling with ^89^Zr and ^225^Ac

N4MU01 was conjugated with *p*-SCN-Bn-DFO for labeling with ^89^Zr followed by quality control as described previously (16). Radiolabeling of DFO-N4MU01 with ^89^Zr and purification were done as reported previously (16).

The 18-membered macrocyclic bifunctional chelator 6-((16-((6-carboxypyridin-2-yl)methyl)-1,4,10,13-tetraoxa-7,16-diazacyclooctadecan-7-yl)methyl)-4-isothiocyanatopicolinic acid (*p*-SCN-Macropa) was synthesized as reported earlier (17). The conjugation of N4MU01 with SCN-Macropa for the labeling with ^225^Ac was performed following the standard operating procedure (supplemental methods). Quality control was done using size-exclusion chromatography/high-performance liquid chromatography and flow cytometry. Radiolabeling with ^225^Ac was performed as previously reported (16).

The stability of [^89^Zr]Zr-DFO-N4MU01 and [^225^Ac]Ac-Macropa-N4MU01 conjugates at 37°C was investigated using instant thin-layer chromatography by analyzing aliquots of the radiolabel over time as reported previously (details in the supplemental materials) (16).

### Radioligand Binding Assay of [^89^Zr]Zr-DFO-N4MU01 and [^225^Ac]Ac-Macropa-N4MU01

The binding of [^89^Zr]Zr-DFO-N4MU01 to nectin-4–positive MCF-7 cells and [^225^Ac]Ac-Macropa-N4MU01 to nectin-4–positive MDA-MB-468 was determined using a saturation radioligand binding assay as described previously (details in the supplemental materials) (18).

### Small-Animal PET/CT Imaging, Biodistribution, and Pharmacokinetics of [^89^Zr]Zr-DFO-N4MU01

Female CD-1 nude mice (*n* = 4) bearing a nectin-4–positive MDA-MB-468 xenograft and female BALB/c mice (*n* = 4) bearing 4T1._nectin-4_ tumors for syngeneic mouse models were injected intravenously with 12 ± 1 MBq (21–26 µg) of [^89^Zr]Zr-DFO-N4MU01 when the tumor size was at least 150 mm^3^. Additionally, mice bearing an MDA-MB-468 xenograft (*n* = 4) were injected intravenously with 200 µg of unlabeled N4MU01 4 h before the same injection of [^89^Zr]Zr-DFO-N4MU01 to preblock nectin-4 receptors. Small-animal PET/CT imaging was done using the Vector^4^CT (MILabs) scanner at different times. Biodistribution studies involved sacrificing mice at 24 and 120 h after injection, with radioactivity measured in all major organs and blood (details in the supplemental materials).

The pharmacokinetics of [^89^Zr]Zr-DFO-N4MU01 was studied in naïve female CD-1 nude mice (*n* = 3/group) as described previously (details in the supplemental materials) (19). All animal studies were approved by the University of Saskatchewan Animal Care and Use Committee protocol #20220021.

### In Vitro Cytotoxicity

The in vitro cytotoxicity (inhibitory concentration of 50% [IC_50_]) of [^225^Ac]Ac-Macropa-N4MU01, control immunoconjugates anti-CD20 [^225^Ac]Ac-Macropa-rituximab, and unlabeled N4MU01 in nectin-4–positive MDA-MB-468, MCF-7, and nectin-4–negative MDA-MB-231 was determined using Incucyte Cytotox Red reagent in an Incucyte S3 live-cell imager (Essen BioScience) as previously reported (details in the supplemental materials) (18).

### Biodistribution and Dosimetry of [^225^Ac]Ac-Macropa-N4MU01

To estimate the radiation dose to healthy tissues, naïve BALB/c nude mice (*n* ≥ 4/group) were administered 13 kBq of [^225^Ac]Ac-Macropa-N4MU01 via the tail vein and sacrificed at 1, 24, 120, and 264 h after injection followed by biodistribution studies. The mouse biodistribution percent of injected activity per gram (%IA/g) data were extrapolated to human data (%IA) using the formula %IA (human) = %IA/g (mouse) × total body weight of mouse (in kg) × mass of (female) human organ (in g)/total body weight of (female) human (in kg). For each organ, this was plotted against sampling time and used to obtain an estimate of the residence time of the agent in the organ in MBq·h/MBq, represented by the area under the time–activity function integrated to infinity (complete decay) of the ^225^Ac. The residence time was fitted into the OLINDA kinetics model (OLINDA/EXM version 2.2; Hermes Medical Solutions) to generate absorbed doses in units of mSv/MBq of ^225^Ac administered.

### Safety of [^225^Ac]Ac-Macropa-N4MU01

Safety studies were conducted on 6-wk-old female naïve BALB/c mice (*n* = 4–5 per group) from Charles Rivers. Acute (day 2) and delayed (day 10) toxicity was studied after a single injection of radiopharmaceutical, whereas chronic (day 20) toxicity was studied after repeated dosage. Safety was studied after a tail vein injection of a single dose or 2 doses of [^225^Ac]Ac-Macropa-N4MU01 (1.5 µg, 15 kBq). A detailed description is available in the supplemental materials.

### Radioimmunotherapy

In vivo efficacy studies were done using athymic nude mice and immune-competent BALB/c mice aged 4–6 wk bearing MDA-MB-468 and 4T1._nectin-4_ tumors, respectively. All the experiments and euthanasia were performed in accordance with University Animal Care Committee guidelines of the University of Saskatchewan. Mice bearing MDA-MB-468 xenografts were divided into 4 groups (*n* ≥ 4/group), namely, [^225^Ac]Ac-Macropa-N4MU01 (2 doses of 13 kBq/dose), [^225^Ac]Ac-Macropa-N4MU01 (2 doses of 18.6 kBq/dose), saline treatment, and unlabeled N4MU01. Mice bearing 4T1._nectin-4_ tumors were divided into 4 groups (*n* ≥ 5/group), namely, [^225^Ac]Ac-Macropa-N4MU01 (2 doses of 13 kBq/dose), therapeutic dose of N4MU01 (2 doses of 100 μg/dose), N4MU01 (2 doses of 1.3 μg/dose), and saline control. The treatment of mice started at an average tumor volume of 50.9 ± 32.6 mm^3^ (MDA-MB-468) and 56.6 ± 32.2 mm^3^ (4T1._nectin-4_). All treated mice received 2 treatment doses via the tail vein on days 0 and 10. Tumor growth was monitored by measuring the greatest length and width using a digital caliper (tumor volume = (length × width^2^)/2). The study was terminated when the tumors reached a volume of more than 1,500 mm^3^. These volumes were used to determine survival in the different groups using Kaplan–Meier curves. The body weight of each mouse was recorded during the experimental period.

### Statistical Analysis

All data are expressed as the mean ± SD or mean ± SE of at least 3 independent experiments. A 2-tailed Student *t*-test or ANOVA with a Bonferroni post hoc test was used to assess the statistical significance between the groups. All graphs were prepared and analyzed using GraphPad Prism (version 9) and a *P* value of less than or equal to 0.05 was considered significant.

## RESULTS

### In Vitro Characterization of N4MU01

**FIGURE 1.**
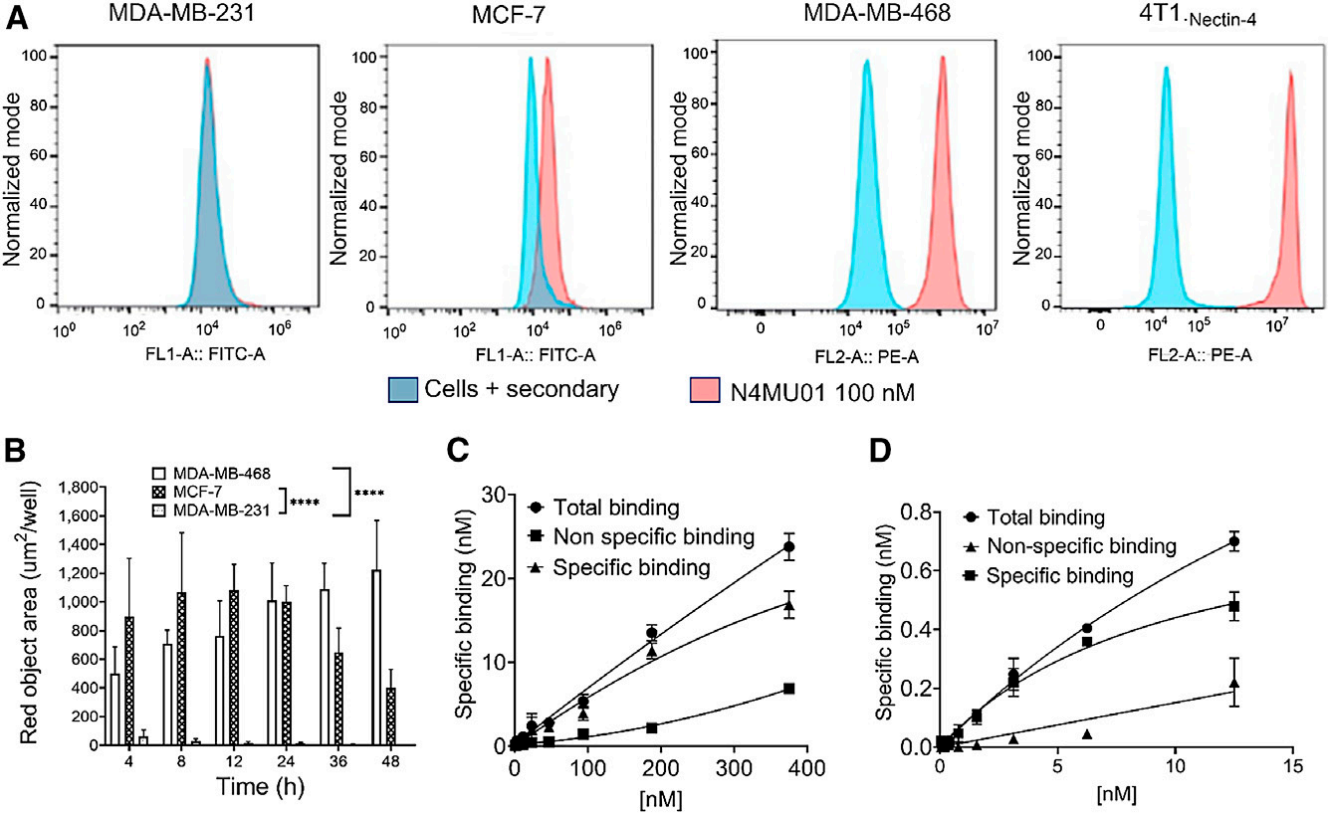
Flow cytometry binding, internalization, and radioligand binding assay of anti–nectin-4 antibody (N4MU01) and its conjugates with (A) MDA-MB-231, MCF-7, MDA-MB-468, and 4T1._nectin-4_ cells. Dose-dependent binding was observed in nectin-4–expressing cell lines, MDA-MB-468, MCF-7, and 4T1._nectin-4_. No binding was observed in MDA-MB-231 control cell line. (B) Internalization of N4MU01: MDA-MB-468, MCF-7, and MDA-MB-231 cells were treated with Incucyte FabFluor-labeled N4MU01 (4 μg/mL); High-definition phase and red fluorescence images (×10 magnification) were captured every 2 h for 48 h. All data are shown as mean ± SEM (*n* = 3), and average internalization values for MCF-7 and MDA-MB-468 were compared with that of MDA-MB-231 using ANOVA. Estimation of *K*_D_ values using radioligand binding assay for (C) [^89^Zr]Zr-DFO-N4MU01 in nectin-4–positive MCF-7 and (D) [^225^Ac]Ac-Macropa-N4MU01 in nectin-4–positive MDA-MB-468 cells. ****P < 0.0001. FITC-A = fluorescein isothiocyanate area; FL1-A = fluorescence intensity from channel 1 (green) area; FL2-A = fluorescence intensity from channel 2 (orange) area; PE-A = phycoerythrin area.

In vitro characterization using flow cytometry was performed to determine the binding of N4MU01 to nectin-4–positive MDA-MB-468, MCF-7, and 4T1._nectin-4_ cells (Fig. 1) and nectin-4–negative MDA-MB-231 cell lines. Dose-dependent specific binding was observed with MDA-MB-468, MCF-7, and 4T1._nectin-4_ cells but not MDA-MB-231 cells (Fig. 1A).

A time-dependent increase in red fluorescence was observed in nectin-4–expressing MDA-MB-468 (high expression) and MCF-7 (moderate expression) cell lines but not in the negative control MDA-MB-231 cells at 4–48 h after incubation (Fig. 1B). The N4MU01 showed 72- and 1,100-fold higher internalization in nectin-4–positive MDA-MB-468 cells compared with the negative control MDA-MB-231 cells at 24 and 48 h, respectively. In MCF-7 cells, the N4MU01 showed 71- and 365-fold higher internalization compared with the negative control MDA-MB-231 cells.

### Conjugation and Quality Control of N4MU01 Radioimmunoconjugates

High-performance liquid chromatography analysis showed that the conjugation of *p*-SCN-DFO to N4MU01 resulted in a greater than 99% pure DFO-N4MU01 with no aggregates. The size and purity of DFO-N4MU01 were evaluated using a bioanalyzer (Supplemental Figs. 2A and 2B). Similarly, the conjugation of Macropa to N4MU01 resulted in a greater than 99% pure immunoconjugate with no aggregates (Supplemental Figs. 2C and 2D). Automated electrophoresis using a bioanalyzer showed that DFO-N4MU01 was 90% pure with a molecular weight of 153.2 kDa (vs. 150.2 kDa for unconjugated N4MU01). This indicates that there were 4.0 DFO chelator molecules per antibody molecule.

An in vitro saturation binding assay of unconjugated N4MU01 and DFO-N4MU01 was done using nectin-4–positive MCF-7 cells using flow cytometry. The estimated dissociation constant (*K*_D_) values for unconjugated N4MU01 and DFO-N4MU01 were 2.9 and 3 nM, respectively. The estimated half-maximal effective concentration values for unconjugated N4MU01 and DFO-N4MU01 were 7.3 and 20.0 nM, respectively (Supplemental Fig. 3).

N4MU01 conjugated with DFO or Macropa was labeled with ^89^Zr or ^225^Ac, respectively. The radiochemical yield of [^89^Zr]Zr-DFO-N4MU01 was more than 90% at a specific activity of 0.5 MBq/μg. Similarly, the radiochemical yield of [^225^Ac]Ac-Macropa-N4MU01 was more than 95% at a specific activity of 10 kBq/μg (Supplemental Figs. 2C and 2D). The stability of [^89^Zr]Zr-DFO-N4MU01 and [^225^Ac]Ac-Macropa-N4MU01 was determined at different time points at 37°C in phosphate-buffered saline and human serum using instant thin-layer chromatography. More than 95% of [^89^Zr]Zr-DFO-N4MU01 remained intact for 96 h in human serum and for 72 h in phosphate-buffered saline. Similarly, more than 96% of [^225^Ac]Ac-Macropa-N4MU01 remained intact for 120 h in human serum and was 91% stable in phosphate-buffered saline (Supplemental Fig. 4).

A saturation radioligand binding assay using [^89^Zr]Zr-DFO-N4MU01 on MCF-7 cells and [^225^Ac]Ac-Macropa-N4MU01 on MDA-MB-468 cells showed a dose-dependent increase in specific binding (Figs. 1C and 1D). The estimated *K*_D_ of [^89^Zr]Zr-DFO-N4MU01 was 10 nM, which is a 3.4-fold lower affinity than that with unlabeled N4MU01 and a 3.3-fold lower affinity than that with DFO-N4MU01. The estimated *K*_D_ of [^225^Ac]Ac-Macropa-N4MU01 was 14.8 nM, which is a 3.7-fold lower affinity than that with unlabeled N4MU01 and similar to that of Macropa-N4MU01.

### Pharmacokinetics, Biodistribution, and Small-Animal PET/CT of [^89^Zr]Zr-DFO-N4MU01

**FIGURE 2.**
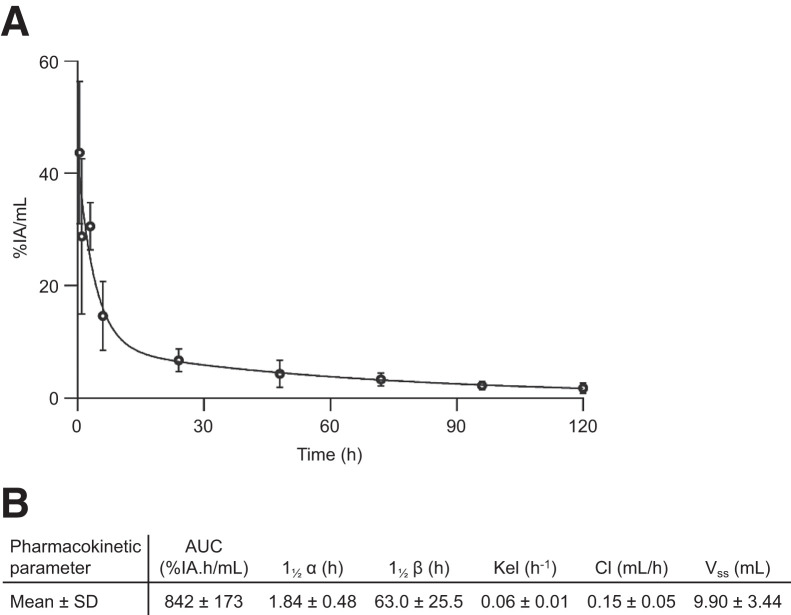
Blood pharmacokinetic of [^89^Zr]Zr-DFO-N4MU01 in healthy CD-1 nude mice. [^89^Zr]Zr-DFO-N4MU01 showed biphasic *t*_1/2_ (A) with fast distribution and moderate elimination (B). AUC = area under curve; Cl = systemic clearance; Kel = elimination constant; V_ss_ = volume of distribution at steady state.

The pharmacokinetic profile of [^89^Zr]Zr-DFO-N4MU01 injected in CD-1 nude mice showed a fast distribution half-life (*t*_1/2α_) of 1.84 ± 0.48 h and a moderate elimination *t*_1/2β_ of 63.0 ± 25.5 h (Fig. 2).

**FIGURE 3.**
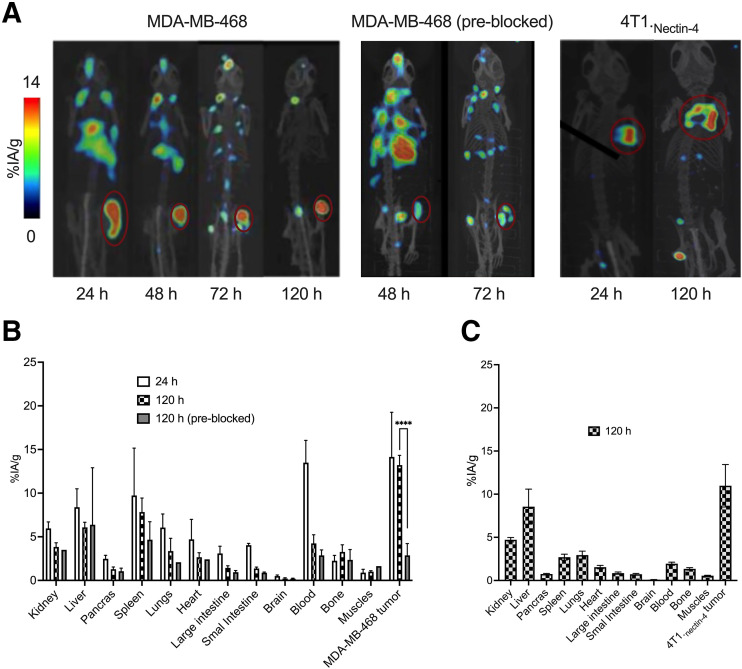
Small-animal PET/CT imaging and biodistribution of [^89^Zr]Zr-DFO-N4MU01. (A) Maximum-intensity projection small-animal PET/CT images of unblocked, N4MU01 (200 µg) preblocked CD-1 nude mice bearing nectin-4–positive MDA-MB-468 xenografts (right flank) and syngeneic mouse model bearing 4T1._nectin-4_ tumors at different time points after injection of 12 MBq of [^89^Zr]Zr-DFO-N4MU01. (B) Biodistribution of [^89^Zr]Zr-DFO-N4MU01 in nectin-4–expressing MDA-MB-468 xenografts and preblocked MDA-MB-468 xenografts. (C) Biodistribution in female BALB/c mice bearing 4T1._nectin-4_ tumors at 120 h after injection. ****P < 0.0001.

Small-animal PET/CT imaging experiments were conducted with [^89^Zr]Zr-DFO-N4MU01 in mice bearing an MDA-MB-468 xenograft and in the syngeneic mouse model bearing 4T1._nectin-4_ tumors. High tumor uptake was delineated in nectin-4–positive xenografts, as shown by small-animal PET/CT imaging at 24–120 h after injection (Fig. 3A). There was a visibly low tumor uptake of [^89^Zr]Zr-DFO-N4MU01 when preblocked with unlabeled antibody (Fig. 3A). For the syngeneic mouse model, tumor uptake increased over time, as shown by the small-animal PET/CT imaging at 24 and 120 h after injection (Fig. 3A).

Biodistribution of [^89^Zr]Zr-DFO-N4MU01 was performed at 24 and 120 h after injection (Fig. 3B) in female CD-1 nude mice bearing nectin-4–positive MDA-MB-468 xenografts. To investigate the specificity, we preblocked the mice bearing MDA-MB-468 xenografts using 200 µg of unlabeled N4MU01 4 h before injection of [^89^Zr]Zr-DFO-N4MU01. In CD-1 nude mice, tumor uptake of MDA-MB-468 was similar at 24 h (14.1 ± 5.1 %IA/g) and 120 h after injection (13.2 ± 1.1 %IA/g) (*P* > 0.9999). Preblocking with unlabeled N4MU01 reduced tumor uptake to 2.8 ± 1.3 %IA/g at 120 h (*P* < 0.0005) after injection. There was a slightly high uptake of [^89^Zr]Zr-DFO-N4MU01 in the lungs (6.0 ± 1.5% IA/g) and heart (4.7 ± 2.3 %IA/g) at 24 h after injection, likely reflecting blood-pool activity. However, this decreased to 3.4 ± 1.5 %IA/g and 2.7 ± 0.5 %IA/g at 120 h after injection, respectively. The tumor-to-blood ratio for MDA-MB-468 was 1.0 at 24 h and 3.1 at 120 h after injection. The tumor-to-muscle ratio for MDA-MB-468 was 13.2 compared with 1.7 for preblocking at 120 h after injection.

Biodistribution of [^89^Zr]Zr-DFO-N4MU01 in mice bearing 4T1._nectin-4_ tumors was performed at 120 h after injection (Fig. 3C), and the tumor uptake was 11 ± 2.5 %IA/g. At 120 h after injection, the tumor-to-muscle ratio was 20 and tumor-to-blood ratio was 5.5, which was 0.7- and 0.6-fold higher than that observed in the immune-deficient nude mice. Spleen uptake was 2.7 ± 2.5 %IA/g, which was 3.7-fold lower compared with the spleen uptake observed in the immune-deficient nude mice.

### In Vitro Cytotoxicity of [^225^Ac]Ac-Macropa-N4MU01

Live-cell imaging was used to study the in vitro cytotoxicity of unlabeled N4MU01 and [^225^Ac]Ac-Macropa-N4MU01 on MDA-MB-468, MCF-7, and MDA-MB-231 cells (Supplemental Table 1). Phase-contrast images showed potent cytotoxicity with [^225^Ac]Ac-Macropa-N4MU01 compared with unlabeled N4MU01. Increased cytotoxicity was observed in MDA-MB-468 at 24 h using [^225^Ac]Ac-Macropa-N4MU01 with an IC_50_ of 1.2 kBq/mL, whereas unlabeled N4MU01 had no effect on MDA-MB-468 cells. The IC_50_ values for MCF-7 and MDA-MB-231 cells were consistent with moderate and no expression of nectin-4, respectively.

### Biodistribution and Dosimetry of [^225^Ac]Ac-Macropa-N4MU01

The biodistribution of [^225^Ac]Ac-Macropa-N4MU01 was studied in naïve BALB/c mice. The uptake of [^225^Ac]Ac-Macropa-N4MU01 was high in the kidneys, liver, spleen, lungs, and blood at early time points, but this uptake decreased over time. Ten days after injection, high uptake was observed in the spleen (4 ± 1.3 %IA/g), lungs (6 ± 1.6 %IA/g), and the blood (5.8 ± 0.9 %IA/g) (Supplemental Table 2). Human organ dosimetry estimates of [^225^Ac]Ac-Macropa-N4MU01 (Table 1) show the organs receiving the highest dose are the lungs > liver > spleen and the total whole-body dose estimate of 18.9 mSv/MBq.

**TABLE 1.** Projected Human Absorbed Doses (mSv/MBq) of [^225^Ac]Ac-Macropa-N4MU01

Organ	Absorbed dose* (mSv/MBq)
Adrenal gland	8.59 × 10^−1^
Brain	7.03 × 10^0^
Breast	1.29 × 10^−1^
Esophagus	4.33 × 10^−1^
Left colon	1.13 × 10^0^
Small intestine	1.29 × 10^0^
Stomach	6.57 × 10^−1^
Rectum	4.37 × 10^−2^
Heart wall	2.01 × 10^2^
Kidneys	3.43 × 10^2^
Liver	3.02 × 10^2^
Lungs	5.08 × 10^2^
Ovaries	5.76 × 10^−2^
Pancreas	6.81 × 10^1^
Red marrow	1.52 × 10^−1^
Spleen	3.73 × 10^2^
Thymus	4.69 × 10^−1^
Bladder	3.10 × 10^−1^
Uterus	6.64 × 10^−2^
Total body	1.89 × 10^1^
Effective dose	8.24 × 10^1^

* For administration in females.

### Safety of [^225^Ac]Ac-Macropa-N4MU01

Single dose acute (day 2) toxicity was determined in naïve BALB/c mice. At day 2 after injection, there was no significant difference (*P* > 0.05) in the blood chemistry and most of the complete blood count parameters in mice compared with the control group, as detailed in Supplemental Table 3. Similarly, at day 10 after injection, there was no significant difference in the blood chemistry and complete blood count parameters in mice compared with the control group (Supplemental Table 3). Furthermore, at day 20 after injection, there was no significant difference (*P* > 0.05) in the blood chemistry and most of the complete blood count parameters between the treated group and the control group (Supplemental Table 3). Histopathologic examination of necropsy-stained slices showed no damage to the organs after administration of a single or double dose of 15 kBq (Supplemental Figs. 5 and 6).

### Radioimmunotherapy

**FIGURE 4.**
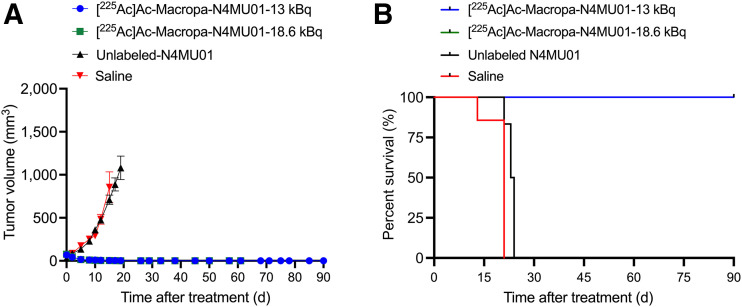
Efficacy of [^225^Ac]Ac-Macropa-N4MU01 in MDA-MB-468 xenograft mouse model. (A) Average tumor volumes of MDA-MB-468 xenografts (*n* = 4–7 mice/group). (B) Kaplan–Meier survival curve of mice bearing MDA-MB-468 xenografts. Tumor-bearing mice (*n* ≥ 4/group) were treated using [^225^Ac]Ac-Macropa-N4MU01 (2 × dose of 13 kBq), [^225^Ac]Ac-Macropa-N4MU01 (2 doses of 18.6 kBq), unlabeled N4MU01 at 10 d apart, or saline control. Study endpoint was considered as tumor volume ≥1,500 mm^3^ or survival for 90 d.

The efficacy of [^225^Ac]Ac-Macropa-N4MU01 against TNBC MDA-MB-468 mouse xenografts was studied. Animals treated with 2 doses of 13 kBq of [^225^Ac]Ac-Macropa-N4MU01 or 18.6 kBq of [^225^Ac]Ac-Macropa-N4MU01 had complete remission, unlike the saline and unlabeled N4MU01 controls (Fig. 4A; Supplemental Fig. 7 for individual mouse tumor growth curves). All the mice in saline group reached the study endpoint with a tumor volume of at least 1,500 mm^3^ by day 21. One of 6 mice (16.6%) treated with unlabeled N4MU01 reached the study endpoint by day 21, and the rest reached the endpoint by day 24. The Kaplan–Meier survival curve showed that mice treated with [^225^Ac]Ac-Macropa-N4MU01 survived for the whole period of the therapy study of 90 d. However, the median survival of the saline group was 21 d, and the median survival of the unlabeled N4MU01-treated mice was 23.5 d (Fig. 4B).

**FIGURE 5.**
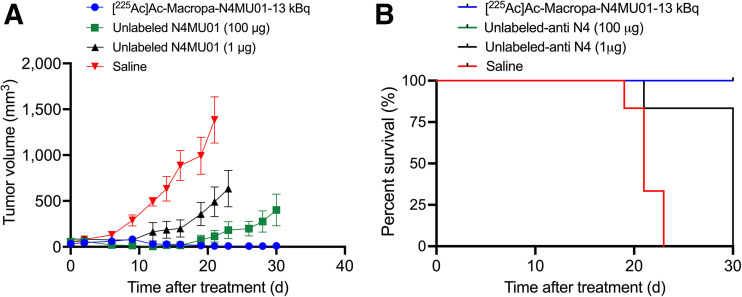
Efficacy of [^225^Ac]Ac-Macropa-N4MU01 in 4T1._nectin-4_ syngeneic mouse model. (A) Average tumor volumes (*n* = 5–6 mice/group). (B) Kaplan–Meier survival curve. Tumor-bearing mice (*n* ≥ 5/group) were treated using [^225^Ac]Ac-Macropa-N4MU01 (2 doses of 13 kBq), therapeutic dose of N4MU01 (2 doses of 100 mg), N4MU01 (2 doses of 1.3 mg) administered at 10 d apart, or saline control. Study endpoint was considered as tumor volume ≥1,500 mm^3^. N4 = nectin-4.

To further assess the efficacy of [^225^Ac]Ac-Macropa-N4MU01, a 4T1._nectin-4_ syngeneic BALB/c mouse model was used (Fig. 5A). The transfection of the mouse breast cancer cell line 4T1 with human nectin-4 was confirmed using flow cytometry (Supplemental Fig. 8). A mixed population of nectin-4–transfected and nontransfected 4T1 cells was observed. Cell sorting using fluorescence-activated cell sorting analysis was performed to isolate the nectin-4–transfected 4T1 population (Supplemental Fig. 9).

For the syngeneic mouse model, 5 of 6 mice (83.4%) treated with 2 doses of 13 kBq of [^225^Ac]Ac-Macropa-N4MU01 had complete remission by day 30. Five of 6 mice (83.4%) treated with the therapeutic dose of unlabeled N4MU01 (100 μg) had delayed tumor growth, whereas 1 of 6 mice (16.6%) had complete remission (Fig. 5A; tumor growth curves of individual mice are shown in Supplemental Fig. 10). All the mice in the saline and the unlabeled N4MU01 groups reached the study endpoint with a tumor volume of at least 1,500 mm^3^ by day 23 and day 30, respectively. The median survival for mice treated with [^225^Ac]Ac-Macropa-N4MU01 and the unlabeled N4MU01 therapeutic dose (100 mg) was not reached by day 30. However, the median survival for the saline group was 21 d (Fig. 5B). There was no apparent toxicity throughout the treatment period, evident from the weight gain (Supplemental Figs. 11A and 11B).

## DISCUSSION

An optimal cancer cell surface biomarker is essential for developing effective targeted therapeutics against TNBC. Nectin-4 is overexpressed in 60%–70% of TNBCs and in TNBC metastases and basal subtypes and is absent in normal epithelial breast tissue (20), making it an ideal target for theranostics. This study describes the preclinical evaluation of a fully human anti–nectin-4 antibody PET imaging probe and radioimmunotherapeutic against TNBC. Although a few groups have evaluated anti–nectin-4 immuno-PET/SPECT imaging, photothermal probes, and ADCs, to the best of our knowledge, no reports of α-particle (^225^Ac) radiotherapeutics of nectin-4 exist in the literature (21,22).

^89^Zr is a nearly ideal PET isotope for imaging biologics. Using N4MU01 (*K*_D_, 3 nM), a [^89^Zr]Zr-DFO-N4MU01 imaging probe was developed. Shao et al. (22) studied the tumor uptake of [^99m^Tc]Tc-HYNIC-mAb_nectin-4_ in a nectin-4–positive MDA-MB-468 xenograft. Maximum tumor uptake in the MDA-MB-468 xenograft was 15.32 ± 1.04 percentage of injected dose per gram (%ID/g) and 4.33 ± 0.48 %ID/g when preblocked, which is similar with this study for the same model. Additionally, the tumor-to-blood and tumor-to-muscle ratios for our study are comparable with those for the [^99m^Tc]Tc-HYNIC-mAb_nectin-4_ tracer. Campbell et al. evaluated ^89^Zr-AGS-22M6 in tumor-bearing mice and cynomolgus monkeys (21). Tumor uptake of [^89^Zr]Zr-AGS-22M6 in nectin-4–transduced MDA-MB-231 cells (MDA-MB-231-nectin-4) transduced to express the receptor ranged from an average of 38.8 ± 2.8 %ID/g on day 1 to an average of 39.9 ± 5.9 %ID/g on day 6 with an average high of 45.3 ± 2.4 %ID/g on day 3 compared with nontransduced receptor–negative MDA-MB-231-neo that ranged from ranged from an average of 16.3 ± 1.8 %ID/g on day 1 to an average of 17.2 ± 1.3 %ID/g on day 6 with an average high of 18.2 ± 2.8 %ID/g on day 2. Similar tumor uptake ratios were found for [^89^Zr]Zr-AGS-22M6 in nectin-4–positive patient-derived xenografts compared with nectin-4–negative patient-derived xenografts. In the current study, the tumor uptake of [^89^Zr]Zr-DFO-N4MU01 in MDA-MB-468 decreased from 13.2 to 2.8 %IA/g when preblocked with cold antibody, which is indicative of better specificity compared with [^89^Zr]Zr-DFO-AGS-22M6 despite its apparent lower *K*_D_ of 0.01 nM (7). In addition, [^89^Zr]Zr-DFO-N4MU01 displayed a fast distribution *t*_1/2α_ of 1.84 h and a relatively moderate clearance *t*_1/2β_ of 63 h compared with other slow-clearing immunoglobulin G such as trastuzumab, which is advantageous for imaging.

Eighteen-membered–ring macrocylic chelator Macropa forms a highly stable and inert complex with ^225^Ac (16,17). To investigate the antitumor effects of the radioimmunoconjugate, the in vitro cytotoxicity was studied using the Incucyte S3 live-cell imaging system. [^225^Ac]Ac-Macropa-N4MU01 displayed enhanced cytotoxicity to nectin-4–expressing cells, whereas unlabeled N4MU01 was not cytotoxic to nectin-4–expressing TNBC MDA-MB-468. The IC_50_ of 1.2 kBq/mL was similar to that of other highly cytotoxic [^225^Ac]Ac-Macropa–labeled radioimmunoconjugates (23,24). Such findings should not be interpreted as a surrogate for tumor growth inhibition.

[^225^Ac]Ac-Macropa-N4MU01 showed very favorable dosimetry in all healthy organs because of its excellent clearance rates from nectin-4–negative healthy tissues. The highest organ dose was observed for the liver, lungs, and spleen. To the best of our knowledge, no organ dose estimates for the anti–nectin-4 radioimmunoconjugate has been reported in the literature for comparison. [^225^Ac]Ac-Macropa-N4MU01 (total body dose of 18.9 mSv/MBq) was almost 1.5-fold less than the widely investigated peptide-targeted radioconjugate [^225^Ac]Ac-DOTA-PSMA-617 (28 mSv/MBq) (25). A 15 kBq dose of [^225^Ac]Ac-Macropa-N4MU01 was well tolerated, as indicated by hematologic, chemistry, and histopathology analyses (Supplemental Fig. 5; Supplemental Table 3).

The effectiveness of unlabeled N4MU01 and [^225^Ac]Ac-Macropa-N4MU01 against the TNBC MDA-MB-468 xenograft was studied. All mice bearing the MDA-MB-486 xenograft treated with saline or unlabeled N4MU01 reached the tumor endpoint of 1,500 mm^3^ by day 21. Significant dose-dependent tumor inhibition was observed for 13 and 18.6 kBq doses at day 28 compared with controls. Additionally, the effectiveness of [^225^Ac]Ac-Macropa-N4MU01 was evaluated in a syngeneic mouse model after transfection of murine 4T1 cells with nectin-4. Of 6 mice bearing 4T1._nectin-4_ cells who were treated using 2 doses of 13 kBq [^225^Ac]Ac-Macropa-N4MU01, 5 had complete remission and the remaining mouse had a tumor volume of 8.2 ± 14.0 mm^3^ at day 28. Untreated mice and mice treated using 1 µg (the equivalent antibody mass dose of the ^225^Ac-labeled agent) reached a tumor volume greater than 1,500 mm^3^ by day 21. Unlike the ADC studies reported by M-Rabet et al. (6) and Challita-Eid et al. (7), tumor regrowth was not observed using [^225^Ac]Ac-Macropa-N4MU01.

## CONCLUSION

Here, the development of the first anti–nectin-4 α-particle therapeutic agent is reported. N4MU01 antibody is fully human with less immunogenicity than the murine-based antibodies. The high specificity and tumor uptake of [^89^Zr]Zr-DFO-N4MU01 and the exceptional therapeutic efficacy of [^225^Ac]Ac-Macropa-N4MU01 warrant further evaluation and potentially clinical translation.

## DISCLSOURE

This work was funded by a Canadian Institute of Health Research (CIHR) project grant (#437660) to Humphrey Fonge and Cancer Research Society grant (CRP-178671) to Maruti Uppalapati and Humphrey Fonge. Maruti Uppalapati, Humphrey Fonge, and Hanan Babeker have filed a patent for this invention. No other potential conflict of interest relevant to this article was reported.

## Notes

KEY POINTS **QUESTION:** Can N4MU01 radiolabeled with ^225^Ac be effective against nectin-4–positive TNBC xenografts? **PERTINENT FINDINGS:** [^225^Ac]Ac-Macropa-N4MU01 was effective in prolonging the survival of mice bearing aggressive nectin-4–positive TNBC xenografts and was safe in healthy mice. **IMPLICATIONS FOR PATIENT CARE:** [^225^Ac]Ac-Macropa-N4MU01 could result in significant survival improvements, which represent 60%–70% of TNBC patients, when and if translated in a clinical trial.
